# Assessment of pancreatic neuroendocrine tumor cytologic genotype diversity to guide personalized medicine using a custom gastroenteropancreatic next-generation sequencing panel

**DOI:** 10.18632/oncotarget.18750

**Published:** 2017-06-28

**Authors:** Ferga C. Gleeson, Jesse S. Voss, Benjamin R. Kipp, Sarah E. Kerr, John S. Van Arnam, John R. Mills, Cherisse A. Marcou, Amber R. Schneider, Zheng Jin Tu, Michael R. Henry, Michael J. Levy

**Affiliations:** ^1^ Division of Gastroenterology & Hepatology, Mayo Clinic Rochester, MN, USA; ^2^ Department of Laboratory Medicine-Pathology, Mayo Clinic Rochester, MN, USA; ^3^ Divisions of Biomedical Statics & Informatics, Department of Health Sciences Research, Mayo Clinic Rochester, MN, USA

**Keywords:** pancreas neuroendocrine tumor, endoscopic ultrasound fine needle aspiration, targeted next-generation sequencing, prognostic biomarker, predictive biomarker

## Abstract

**Background:**

Recent genetic studies have highlighted that alterations in MEN1, chromatin remodeling genes, and mammalian target of rapamycin (mTOR) pathway genes are the most frequent molecular events identified in pancreas neuroendocrine tumors (pNETs). The prognostic or predictive impact of these biomarkers and other less frequently observed aberrations, i.e. PTEN, TSC2 and PIK3CA are relatively unknown. The aims of this targeted next generation sequencing (NGS) study were to assess tumor cytology genotype diversity, to survey for potential adverse prognostic biomarkers and the prevalence of mTOR pathway variants.

**Methods:**

Using a custom 15 gene gastroenteropancreatic neuroendocrine tumor panel, targeted NGS of archived (2002-2013) primary pNETs (n=90) and pNET liver metastasis (n=32) cytology smears was performed.

**Results:**

The genetic variant landscape revealed that 21% and 28% of primary and metastatic liver pNETs harbored ≥ 2 variants per tumor, respectively. The most prevalent primary tumor variants were in the MEN1 (42%), DAXX (11%), ATRX (10%), and TSC2 (8%) genes. Patients harboring aberrations in TSC2, KRAS or TP53 were more likely to experience disease progression and reduced overall survival, when compared to individuals who were wild-type. The prevalence of these potential prognostic biomarkers in early disease was observed in 3.3% of the primary tumor cohort. mTOR pathway variants including alterations in PTEN, TSC2 and PIK3CA were identified in 10% and 12.5% of primary tumors and pNET liver metastasis, respectively.

**Conclusion:**

Cytology based tumor genotyping revealed a broad spectrum of genetic variants including possible adverse prognostic biomarkers, reflective of an aggressive phenotype. It also demonstrated the prevalence of potential predictive biomarkers for mTOR pathway inhibitor sensitivity.

## INTRODUCTION

Pancreas neuroendocrine tumors (pNETs) are a heterogeneous collection of rare tumors with a distinct gene mutation spectrum when compared to pancreatic ductal adenocarcinomas (PDAC), with an overall 10-year survival of 47%. [1, 2] A recent whole exome sequencing study of sporadic pNETs identified key molecular alterations at a low frequency, presumably reflecting the indolent nature of this tumor type. [3] The relatively unique genetic variant landscape revealed that 44% of pNETs harbored a *MEN1* (a tumor suppressor gene) alteration and 43% either a *DAXX* or *ATRX* gene (chromatin remodeling complex) aberration. Of note, 14% had a mutation in one of the mammalian target of rapamycin (mTOR) pathway genes: *TSC2* (8.8%), *PTEN* (7.4%) and *PIK3CA* (1.4%), thereby providing a potential cohort for targeted therapy.

*MEN1* is thought to play an early role in tumorigenesis as it regulates cellular proliferation. [4] Inactivating *DAXX/ATRX* genetic variants are associated with alternative lengthening of telomeres (ALT), a telomerase-independent homologous recombination-associated process that lengthens telomeres allowing neoplastic cell immortalization. [5] They have been observed in well differentiated, but not in poorly differentiated pNETs. [6] In an immunohistochemistry study, DAXX/ ATRX loss was observed in 26% and 52% of resectable and metastatic unresectable tumors, respectively. [7] Such a loss has been associated with chromosomal instability and may provide important prognostic information. [8] In contrast, DAXX/ATRX wild-type tumors can harbor *TP53*, *KRAS* and *RB* alterations which are associated with poorly differentiated pNETs and a more aggressive phenotype. [6] Genetic signatures may allow for the future development of a biomarker stratification strategy to classify pNETs and direct precision medicine in the future.

Endoscopic ultrasound fine needle aspiration (EUS FNA) is now routinely performed to diagnose and enhance the staging of pNETs, providing a diagnostic sensitivity and specificity of 99% and 100%, respectively. [9–11] Our group has previously demonstrated the potential critical prognostic and theranostic role of EUS with cytology acquisition in the field of molecular medicine for several other tumor types. We have utilized either commercial or custom designed targeted next generation sequencing (NGS) panels to reveal the genetic variant landscape in PDAC, gastrointestinal stromal tumors (GIST’s), rectal and lung cancer. [12–18] Collectively, this work has highlighted the potential role of EUS to provide personalized patient care, due to the enhanced ability to identify prognostic and predictive disease biomarkers, particularly in the field of targeted gene therapy. Our prior work and that of others, has highlighted the difficulty in conducting molecular studies given the absence of dedicated disease specific panels, relying instead on various large more comprehensive expensive platforms that do not lend themselves to disease specific testing. The presented study was largely directed to further develop this clinical need.

The objectives therefore of this translational pNET study that included patients with a spectrum of Stage I-IV disease was to: 1.) Develop a custom designed gastroenteropancreatic targeted NGS gene panel, 2.) Define the genetic variant landscape, 3.) Detect potential adverse prognostic biomarkers, 4.) Determine the prevalence of such adverse prognostic biomarkers in stage I disease, and 5.) Detect the prevalence of mTOR pathway variants.

## RESULTS

### Primary pNET cohort (clinical and tumor demographics)

The pNET EUS FNA cytology smear cohort was comprised of 90 patients[61.1 ± 12.7 years, male gender n=54 (60%)], 85 (94.4%), 87 (96.7%), and 89 (98.9%) of whom, were well-differentiated, sporadic, and non-functioning tumors, respectively. The ENET stages were as follows: T1N0M0 n=50 (55.6%), T2N0M0 n=25 (27.8%), T3N0M0 n=4 (0.04%), T4N0M0 n=7, and T4N0M1 n=4: T4 lesions n=11 collectively (12.2%). The pancreatic tumor locations were as follows: head-uncinate n=21 (23.2%), neck n=8 (8.9%), body n=21 (23.3%), tail n=39 (43.3%) and resection bed n=1 (1.1%) with a median long axis diameter of 14mm (10-25) at EUS.

Patients who proceeded to oncologic surgery [n=42 (46.7%)] underwent a distal pancreatectomy n= 30 (71.4%), a pancreaticoduodenectomy (Whipple procedure) n=4 (9.5%), pNET enucleation n= 4 (9.5%), and a liver resection, small bowel resection, completion pancreatectomy, or a central pancreatectomy, n=1 (2.4%) each, respectively.

Disease progression was experienced by 17 (18.9%) patients with disease metastasis to the liver (n=14), lung (n=2), and retroperitoneal lymph nodes (n=1) at a median time of 0.8 years (0.3-2.5) following EUS FNA. Disease related mortality and overall mortality was experienced by 11 (12.2%) and 21 (23.3%) patients, respectively.

### pNET liver metastasis cohort (clinical and tumor demographics)

This specific cohort was comprised of 32 patients[59.3 ± 13.2 years, male gender n=18 (56.3%)], 26 (81.3%), 31 (96.9%) and 20 (62.5%) of whom were well-differentiated tumors, sporadic and non-functioning, respectively. The functioning primary lesions had elevations in serum gastrin n=4, glucagon n=5; and insulin, vasoactive intestinal polypeptide (VIP), and human pancreatic polypeptide (HPP) in one patient each, respectively. The corresponding primary tumors (n=26 with available information) were located in the head-uncinate n=6 (23.1%), neck n=1 (3.8%), body n=7 (26.9%), and tail n=12 (46.2%) with a median pNET size of 41mm (35-70) upon initial presentation. Disease related mortality and overall mortality for this subgroup was experienced by 20 (62.5%) patients at 3.9 (1.2-6.9) years each from the time of biopsy proven liver metastasis.

### Cytology genotyping using a custom gastroenteropancreatic 15 gene panel

Targeted NGS sequencing revealed 86 genetic variants among the 90 primary pNETS, and 31 variants in 32 pNET liver metastasis, respectively. Thirty-six (40%) and 13 (40.6%) primary tumors and liver metastasis were wild-type (WT) for all assessed 15 genes. Although this WT cohort was older (61.7 ± 13.6 years vs. 56.5 ± 4.9 years; p= 0.0118), there were no differences in gender distribution, presence of a sporadic or functioning tumor, ENET stage, primary tumor size or location, incidence of disease progression or overall survival, when compared to a tumor harboring ≥ 1 genetic variant. Nineteen (21.1%), 8 (8.9%) and 3 (3.3 %) primary tumors and 9 (28.1%), 2 (6.3%) and 1 (3.1 %) liver metastasis lesions harbored ≥ 2, ≥ 3 and ≥ 4 genetic variants per tumor, respectively. There was no relationship between primary tumor size and the number of variants per tumor: correlation coefficient r = 0.076 (95% CI -0.1332 to 0.2787) p = 0.4765.

Genotyping revealed that the majority of the primary tumor variants were identified in MEN1 (42.2%), DAXX (11.1%), ATRX (10%), and TSC2 (7.8%) genes. (Table 1 and Supplementary Table 2-7) The prevalence of TP53 aberrations was significantly greater in liver metastasis as compared to primary tumors (15.6% vs. 3.3%; p=0.0287).

**Table 1:** Genetic variant spectrum and frequency within primary tumors

	GENE	NO. OF VARIANTS IDENTIFIED	NO. OF PATIENTS WITH A VARIANT	% OF PATIENTS WITH A VARIANT
**1.**	MEN1	43	38	42.2
**2.**	DAXX	10	10	11.1
**3.**	ATRX	12	9	10.0
**4.**	TSC2	7	7	7.8
**5.**	KRAS	3	3	3.3
**6.**	TP53	3	3	3.3
**7.**	RB1	2	2	2.2
**8.**	CTNNB1	2	2	2.2
**9.**	RET	1	1	1.1
**10.**	PTEN	1	1	1.1
**11.**	HRAS	1	1	1.1
**12.**	PIK3CA	1	1	1.1
**13.**	NRAS	0	0	0
**14.**	VHL	0	0	0
**15.**	BRAF	0	0	0

One 36 year old male who at EUS had a T4N0M1 25mm poorly differentiated pancreas tail tumor, harbored 5 genetic variants [4 pathogenic and 1 variant of unknown significance (VUS)] with synchronous liver metastases (MEN1 c.1192C>T, p.Gln398*, TP53 c.577C>T, p.His193Tyr, and three ATRX variants: ATRX c.6835G>T, p.Glu2279*, ATRX c.6825G>A, p.Trp2275* and ATRX c.2893G>A, p.Ala965Thr).

### MEN1 - DAXX - ATRX primary tumor genetic variant profiles

All MEN1 variants (n=43) were pathogenic alterations and were composed of the following: n=15 (34.9%) frameshift mutation; n=14 (32.6%) stop gained mutation; n=9 (20.9%) missense variants; n=3 (6.9%) splice site mutations; n=1 (2.3%) in frame deletion; and n=1 (2.3%) start loss. DAXX variants (n=10) were composed of n=7 (70%) frameshift mutations; n=2 (20%) missense variants; and n=1 (10%) nonsense mutation. The ATRX alterations (n=12) were determined to be: n=6 (50%) missense variants; n=4 (33.3%) nonsense mutation; n=1 (8.3%) frameshift mutation; and n=1 (8.3%) splice site mutation.

**Figure 1:**
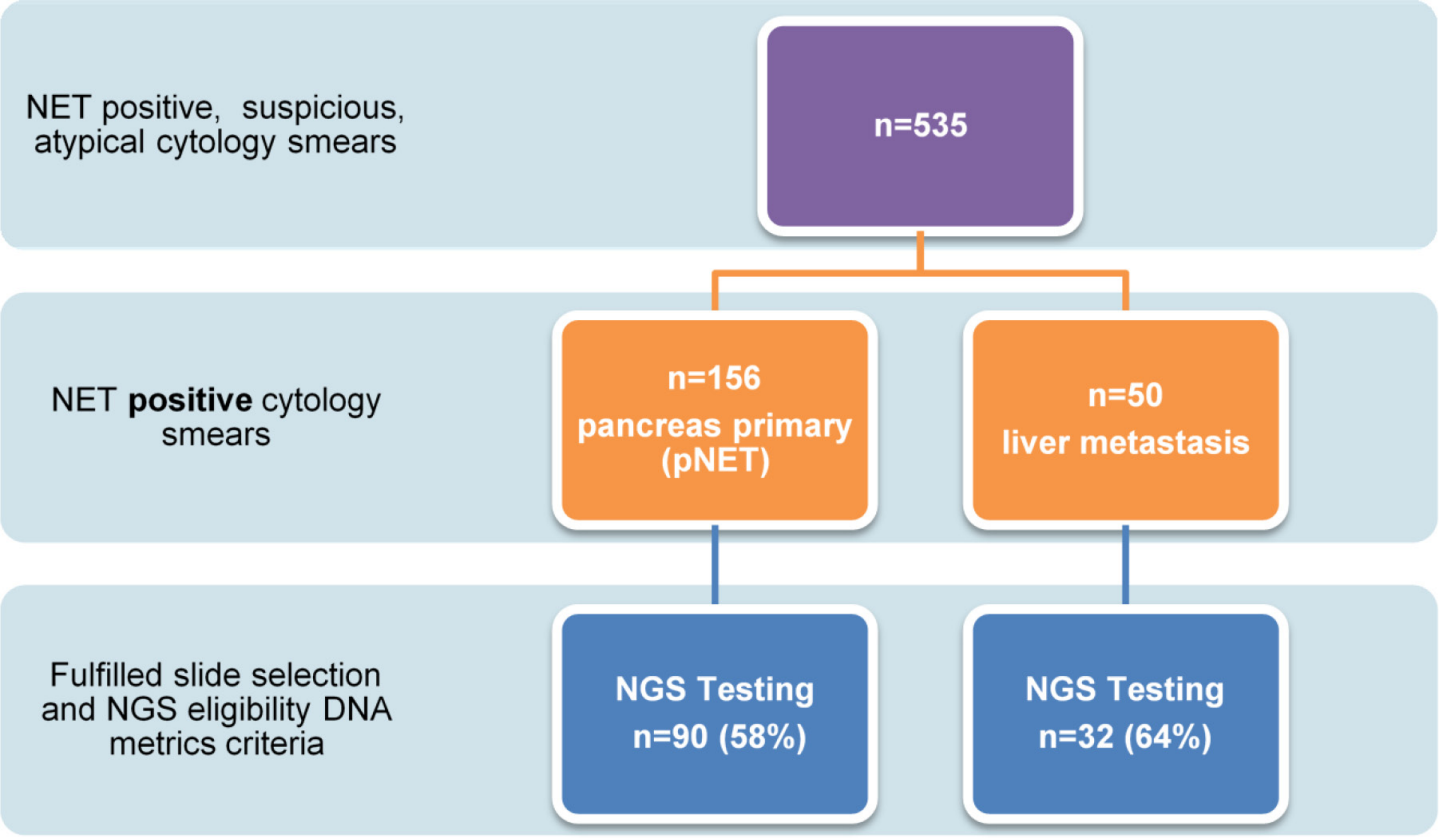
Flow chart to highlight pancreas and liver cytology smear acquisition to completion of targeted NGS Only 59% of archived cytology (2002-2013) single slide smear specimens ultimately fulfilled selection criteria reflecting the need to determine molecular adequacy in addition to diagnostic cellular adequacy to have the ability to complete potential diagnostic, prognostic and theranostic neoplasia assessments.

MEN1 with DAXX or MEN1 with ATRX concurrent variants were present in 6 (6.7%) and 5 (5.6%) patients, respectively. (Figure 1 Supplementary Figure 1) DAXX and ATRX variants were mutually exclusive. A tumor harboring a MEN1 variant was a smaller sized lesion when compared to MEN1 WT lesions: 15.9 ± 12.5 mm vs. 24.0 ± 21.1mm; p=0.0377. Tumors revealing either a DAXX or ATRX variant were larger lesions at diagnosis (28.3 ± 17.6mm vs. 18.7 ± 18.1mm; p=0.0415) and more likely to be ENET stage ≥ T3N0M0 [9 (47.4%) vs. 6 (8.5%); p=0.0003] when compared to DAXX/ ATRX WT. For tumors exhibiting MEN1, DAXX or ATRX alterations, there were no differences in age, gender, presence of a sporadic or functioning tumor, ENET stage, lesion size or location, disease progression or overall survival, when compared to the corresponding WT tumor population.

### Characterization of the stage based upon variant profile

Following AJCC/ ENETS group staging, there were significant differences in the distribution of RET, HRAS, MEN1, DAXX and ATRX variants. MEN1 variants were more frequently present in early disease stage in contrast to DAXX or ATRX, which were observed more frequently in advanced disease stages. (Table 2).

**Table 2:** Disease stage based distribution of tumor clinical and genetic variant characteristics

	STAGE I N = 50 PATIENTS	STAGE II N = 29 PATIENTS	STAGE III N = 11 PATIENTS	STAGE IV N = 32 PATIENTS	P VALUE
AGE	62.04 ± 12.06	61.04 ± 13.2	57.6 ± 12.3	59.3 ±13.2	0.4056
GENDER (MALE)	28 (56%)	18 (62.1%)	8 (72.7%)	18 (56.3%)	0.7396
SPORADIC TUMOR	48 (96%)	28 (96.6%)	11 (100%)	31 (96.9%)	0.9276
NON-FUNCTIONING TUMOR	50 (100%)	28 (96.6%)	11 (100%)	20 (62.5%)	0.0001
NET SIZE (MM)	10.6 ± 3.5	30.1 ± 11	43.9 ± 37.7	^*^53.9 ± 25.5	0.0001
LOCATION (TAIL)	25 (50%)	12 (41.4%)	2 (18.2%)	12 (46.2%)	0.2758
DISEASE PROGRESSION	6 (12%)	4 (13.8%)	7 (63.6%)	20 (62.5%)	0.0001
DISEASE RELATED MORTALITY	3 (6%)	3 (10.3%)	5 (45.5%)	20 (62.5%)	0.0001
VARIANTS PER TUMOR	0.82 ± 0.78	0.82 ± 1.06	2 ± 1.6	0.97 ± 1.05	0.0950
GENETIC VARIANT ≥ 2 PER TUMOR	8 (16%)	6 (20.7%)	6 (54.5%)	2 (6.3%)	0.0769
**GENETIC VARIANTS**	**Stage I** **N = 50 patients**	**Stage II** **N = 29 patients**	**Stage III** **N = 11 patients**	**Stage IV** **N = 32 patients**	
RET	0	0	1 (9.1%)	0	0.0171
PTEN	0	1 (3.4%)	0	2 (6.3%)	0.3114
HRAS	0	0	1 (9.1%)	0	0.0171
MEN1	28 (56%)	6 (20.7%)	4 (36.4%)	11 (34.4%)	0.0492
KRAS	1 (2%)	2 (6.9%)	0	2 (6.3%)	0.5756
RB1	2 (4%)	0	0	0	0.4029
TSC2	2 (4%)	2 (6.9%)	3 (27.3%)	2 (6.3%)	0.0636
TP53	1 (2%)	1 (3.4%)	1 (9.1%)	6 (18.8%)	0.0873
VHL	0	0	0	1 (3.1%)	0.4176
CTNNB1	0	2 (6.9%)	0	0	0.0889
PIK3CA	0	0	1 (9.1%)	0	0.0171
DAXX	2 (4%)	3 (10.3%)	5 (45.5%)	7 (21.9%)	0.0016
ATRX	2 (4%)	5 (17.2%)	2 (18.2%)	1 (3.1%)	0.0219

^*^only 23 cases for primary pNET size identified.

°No NRAS, BRAF variants detected and therefore not in table.

### Prognostic biomarkers for disease progression

**Figure 2:**
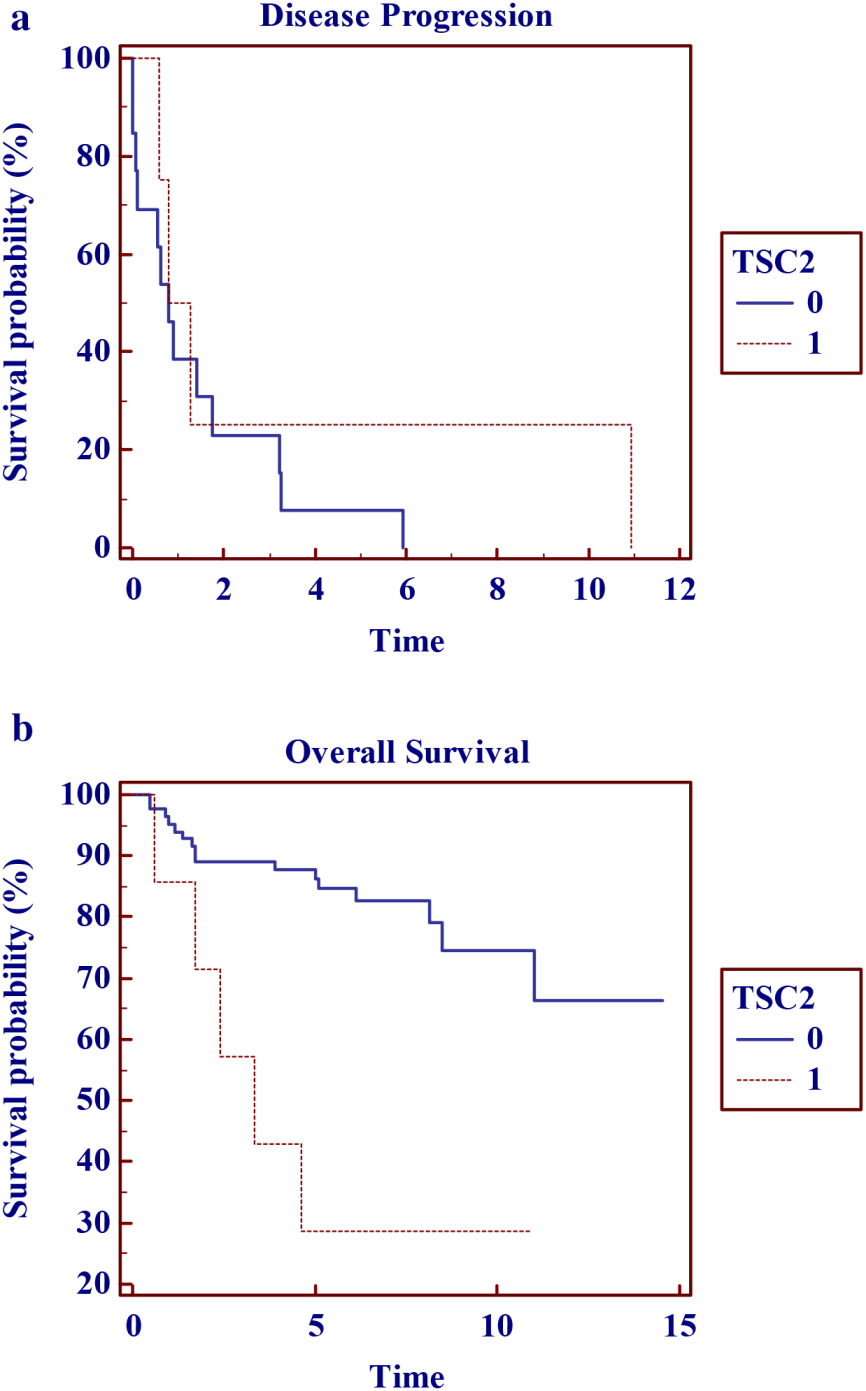
Kaplan Meier Survival curves for tumors harboring TCS2 variants: **(a)** disease progression and **(b)** overall survival in years.

When evaluating tumors with a specific gene alteration frequency of > 3%, patients harboring tumor genetic alterations in TSC2, KRAS or TP53 were more likely to experience disease progression and reduced survival when compared to individuals who were WT for these specific genetic variants. (Figure 2 and Table 3) These potential adverse prognostic biomarkers were observed in early stage disease (EUS ENET TNM Stage T1) in three male patients (6% of lesions < 20mm), each with well-differentiated, sporadic, non-functioning disease or 3.3% of the entire primary tumor cohort. (Table 4 and Supplementary Table 8) Only one pNET patient harbored a combination of TSC2 (c.400G>A, p.Glu134Lys), TP53 (c.818G>A, p.Arg273His), and KRAS (c.35G>C, p.Gly12Ala) pathogenic alterations.

**Table 3:** Disease progression and overall survival among patients with a variant prevalence >3%

	OVERALL SURVIVAL (YEARS)			DISEASE PROGRESSION		OVERALL SURVIVAL	
	Variant frequency	Variant present	Variant Wild Type	HR (95% CI)	P Value	HR (95% CI)	P Value
**MEN1**	42.2%	5.6 (4.2-7 .9)	6.4 (3.8-4.6)	0.77(0.29-2.03)	0.5895	1.44 (0.60-3.40)	0.4269
**DAXX**	11.1%	5.3 (4.1- 8.2)	6.2 (3.9-4.2)	0.36 (0.07-1.76)	0.0627	0.71 (0.176-2.84)	0.5765
**ATRX**	10%	7.1 (4.9-11.2)	5.6 (3.9-8.1)	0.93 (0.20-4.23)	0.9173	1.28 (0.3408-4.80)	0.7391
**TSC2**	7.8%	3.4 (1.7-10.6)	6.2 (4.1-8.2)	0.24 (0.04-1.58)	0.0072	0.21 (0.035-1.24)	0.0008
**KRAS**	3.3%	0.97 (0.6 -6.2)	6.2 (4.0-8.2)	0.094 (0.0031-2.86)	0.0001	0.097 (0.003-2.97)	0.0001
**TP53**	3.3%	0.97 (0.6-1.6)	6.2 (4.1-8.3)	0.041(0.00024-6.85)	0.0001	0.053 (0.0005-5.25)	0.0001

^*^ Insufficient data for statistical analysis of NRAS, PTEN, VHL, and BRAF variants.

∞RET, HRAS, RB1, CTNNB1 and PIK3CA also excluded as a respective prevalence was < 3% each.

**Table 4:** T1N0M0 clinical characteristics, variant distribution and presence of prognostic biomarkers

	EUS ENET TNM STAGE T1 N = 50 PATIENTS	EUS ENET TNM STAGE T2-T4 N = 40 PATIENTS	P VALUE
AGE	62.04 ± 12.06	59.5 ± 13.3	0.3455
GENDER (MALE)	28 (56%)	26 (65%)	0.5163
SPORADIC TUMOR	48 (96%)	39 (97.5%)	0.9
NON-FUNCTIONING TUMOR	50 (100%)	39 (97.5%)	0.4444
SIZE (MM)	10.6 ± 3.5	33.5 ± 21.5	0.0001
LOCATION (TAIL)	25 (50%)	14 (45%)	0.2000
DISEASE PROGRESSION	6 (12%)	11 (27.5%)	0.1022
TIME TO PROGRESSION (YRS)	3.47 ± 4.49	4.39 ± 4.97	0.3596
OVERALL MORTALITY	9 (18%)	12 (30%)	0.2151
DISEASE RELATED MORTALITY	3 (6%)	8 (20%)	0.0561
VARIANTS PER TUMOR	0.82 ± 0.78	0.97 ± 1.16	0.4666
WT FOR ALL 15 GENES	18 (36%)	18 (45%)	0.3973
VARIANT ≥ 1 PER TUMOR	32 (64%)	22 (55%)	0.3973
VARIANT ≥ 2 PER TUMOR	8 (16%)	12 (30%)	0.1358
VARIANT ≥ 3 PER TUMOR	3 (6%)	5 (12.5%)	0.4584
GENETIC VARIANTS^*^			
RET	0	1 (2.5%)	0.4444
PTEN	0	1 (2.5%)	0.4444
HRAS	0	1 (2.5%)	0.4444
MEN1	28 (56%)	10 (25%)	0.0050
KRAS	1 (2%)	2 (5%)	0.5829
RB1	2 (4%)	0	0.5006
TSC2	2 (4%)	5 (12.5%)	0.2345
TP53	1 (2%)	2 (5%)	0.5829
CTNNB1	0	2 (5%)	0.1948
PIK3CA	0	1 (2.5%)	0.4444
DAXX	2 (4%)	8 (20%)	0.0208
ATRX	2 (4%)	7 (17.5%)	0.0721

*NRAS, VHL and BRAF not included in this table as no genetic variants detected.

### Prevalence of mTOR pathway variants

Members of the mTOR pathway to include PTEN, TSC2 and PIK3CA associated genetic variants were identified in 9 (10%) primary pNETs and 4 (12.5%) liver metastasis patients, respectively.

## DISCUSSION

Sporadic and familial pNETs are understudied tumors that follow a hyperplasia to neoplasia sequence. [23] As precision medicine is rapidly becoming the “Holy Grail” to customize individual patient care, little is known of their cytologic genetic signature and how that may deliver prognostic or predictive biomarkers for disease management. Research efforts have been challenging as access to tissue from a broad spectrum of disease stages and cell lines has been limited which is compounded by the fact that these tumors have alterations at a much lower frequency when compared to PDAC. [24] To help address the limitations of pNET molecular testing, we created a dedicated gastroenteropancreatic neuroendocrine 15 gene NGS panel to evaluate pancreas and liver cytology smears collected over 11 years, from a variety of disease stages, obtained via EUS or percutaneous ultrasound guided FNA. This initial work served as a basis for evaluating our current patient cohort. In our cytologic study, 40% of patients were WT for all 15 genes but did not have differences apart from age at presentation, in clinical or tumor characteristics when compared to tumors with ≥ 1 alteration. We observed a broad spectrum of pathogenic alterations in 12 of the 15 genes which revealed that only 20% of tumors harbored ≥ 2 alterations. This was in keeping with the landmark whole exome sequencing study that reported a similar frequency of MEN1, DAXX and ATRX variants. [3] We found no correlation between the number of variants detected per tumor and the lesion size at diagnosis. Our data suggest that MEN1 alterations are an early event in tumorigenesis and that ATRX/DAXX variants may be later events in disease progression or occur more frequently in patients presenting with metastatic disease. Our small cohort of 90 patients may be considered to be a limitation but pNETs are an infrequently assessed tumor.

A multifaceted approach is required to enhance the classification of pNETs to encompass the spectrum of important NET prognostic metrics including tumor morphology, stage, grade and genotype. Tumors harboring changes in TSC2, KRAS and TP53 were more likely to undergo disease progression and had reduced overall survival. Of particular note, these possible aggressive phenotype aberrations were discovered in 6% of stage I disease patients. Given that these genetic alterations were infrequent, we are therefore cautious to pose any firm conclusions. However, EUS detection of poor prognostic molecular markers from early stage (<20mm) and/or well differentiated pNETs could substantially alter management for a cohort of patients in whom conservative non-operative care may be recommended, thus identifying patients with more aggressive tumor phenotypes that would benefit from oncologic resection or early systemic therapy. [25]

Everolimus, an orally administered mTOR inhibitor, provides enhanced outcomes in treating tumors harboring alterations in the mTOR pathway. The findings from the recent RADIANT-4 trial indicate that its use was associated with a significant improvement in progression free survival in patients with advanced, non-functional disease. [26] While the mechanisms of primary or therapy-induced resistance to this targeted therapy are unknown, the evaluation of predicative biomarkers of mTOR pathway sensitivity suggest that Everolimus is potentially beneficial in 10% and 12.5% of our study patients with pancreas and liver NETs, respectively. Our previously established ability to perform genotyping with EUS or US cytology smears provides a potential means of triaging patients into cohorts more likely to respond to agents designed to target specific pathways and thus enhance therapeutic response and patient outcome. Such testing would also have the potential to allow clinicians to avoid the use of the often toxic and expensive therapies in patients unlikely to benefit from such therapy, and those prone to specific drug-induced adverse events.

Pancreas and liver cytology specimens have the potential to capture more mutations due to the natural concentration of neuroendocrine cells while the residual dense stromal matrix and tumor microenvironment is excluded. However, the ability to perform accurate Ki-67 proliferation indices is limited in this setting, as it is only validated in paraffin embedded tissue sections of substantial size. How cytology smears perform to assess epigenetic alterations, microRNA, mRNA, chromosomal translocations, copy number variants, alternative lengthening of telomeres (ALT) or the tumor microenvironment, is largely unknown. This is a field of future study, to try and understand an individual tumor’s biologic behavior. It begs the question, if our approach to sampling such tumors in the future non-operative setting, should include a more comprehensive sampling technique of cytology smears accompanied by needle cores with rapid on-site evaluation (ROSE) for molecular adequacy metrics. This approach would encompass characterization of the tumor by cellular morphology, Ki-67 index calculation, and genetic signature. In addition, it would permit disease grading using the WHO 2010 classification system. Furthermore, it has the potential to identify what appear to be well-differentiated tumors by WHO grading, that have an unexpectedly “aggressive” molecular profile. [27] This multifaceted cytology approach could also provide predictive information pertaining to potential mTOR inhibitor sensitivity at diagnosis to aid therapeutic decision making. A recent whole-genome sequencing study of primary pNETs defined the genomic events to characterize pathogenesis by identifying point mutations, gene fusions, and evaluating gene expression analysis. [28] If this laboratory technique could be applied to non-operative cytologic material it would greatly enhance our understanding of pNET tumor biology.

In summary, our study has demonstrated the ability to develop a custom designed gastroenteropancreatic targeted NGS gene panel that can be applied to cytology specimens obtained by various routes to include endoscopic ultrasound. This, in turn would allow us to successfully apply this new panel to perform sporadic and familial pNET Stage I-IV genotyping. This method has the ability to characterize an individual’s tumor genetic signature and may in the future yield molecular prognostic biomarkers and predictive biomarkers of mTOR sensitivity. Of even more importance, it is likely that as for other tumor types, such molecular profiling and patient stratification will be become a vital aspect of future efforts to provide individualized and targeted therapy to enhance outcomes for appropriately selected patients.

## MATERIALS AND METHODS

### Patient population

Following Mayo Clinic Institutional Research Board approval, DNA was extracted from archived cytology (2002-2013) single slide smear specimens (n=123 patients). Each slide was selected by a pathologist and had at least 3,000 total nucleated cells and ≥ 20% tumor cells in a background of benign nucleated cells. Targeted NGS was performed yielding satisfactory results in 122 patients (90 pNETs and 32 pNET liver metastasis), respectively (Figure 1). All primary pNETsmears were obtained via EUS FNA. The liver metastasis smears were acquired via percutaneous ultrasound (US) (n=30) or EUS FNA (n=2). Evaluation of the Ki-67 index was not assessed in this study due to the use of cytologic specimens rather than core biopsy specimens and the lack of availability of core biopsies/cell blocks for many cases.[19–21] Therefore, pNET’s were morphologically classified as well differentiated (WHO grade 1 and 2) or poorly differentiated (WHO grade 3) based upon cytologic features. Patients were categorized based upon their European Neuroendocrine Tumor Society Tumor Node Metastasis (ENET TNM) Classification and combined ENET/ American Joint Commission on Cancer (AJCC) Stage. [22] There were no patients in this cohort with the following familial syndromes: von Hippel-Lindau disease (VHL), tuberous sclerosis (TSC) or neurofibromatosis type 1 (NF1).

### DNA extraction process

Cytology smear slides were briefly placed on a hot plate set at 120°C until the coverslip detached. Following rinsing in xylene then 95% ethanol, all cellular material from a single slide per patient was scraped with a sterile razor and placed into 1.5 ml tubes. Cytology smear DNA was isolated using the QIAmp DNA Micro kit (Qiagen Inc, Valencia, CA) and was quantified using the Qubit® dsDNA BR assay kit (Life Technologies, Carlsbad, CA) as per standard protocol. DNA metrics highlighted that each pNET slide and each pNET liver metastasis slide yielded a median of 32.6 (10.6-62.4) ng/ μl and 35.6 (17.8-58.3) ng/ μl of DNA, respectively.

### Deep sequencing of multiplex PCR amplicons

Multiplex PCR was performed by amplifying 10ng of DNA in each of 4 separate PCR reactions using a custom GeneRead& DNAseq Targeted Panel V2 that included 533 amplicons covering 33,887 bases targeting the hotspot regions of 15 genes that are most frequently mutated in gastroenteropancreatic neuroendocrine tumors (Qiagen Inc, Valencia, CA) per protocol. (Supplementary Table 1).

The PCR products underwent library preparation using the TruSeq Nano DNA Sample Preparation Kit (Illumina, San Diego, CA) as recommended by the manufacturer starting with the end repair reaction. Up to 96 samples were pooled equimolar and underwent 1x151bp sequencing on an Illumina HiSeq 2500 instrument using the Rapid SBS 200 cycle v2 Reagent Kit (Illumina, San Diego, CA). Internal laboratory studies demonstrated 5-10% analytical sensitivity for mutant alleles at a minimum of 100X coverage.

### Data analysis

Following sequencing completion on the Illumina NGS instrument, the raw sequence reads were extracted and demultiplexed with Illumina CASAVA (Consensus Assessment of Sequence And Variation) program (version 1.8.2) to generate FASTQ files. Sequence FASTQ files were aligned to human genome build hg19 using the CLC BIO Genomics Server (version 6.0) program to produce BAM files. The alignment files were analyzed by the CLC BIO Genomics Server quality and probability variant detection program within a custom bioinformatics pipeline running on the Linux cluster. The single nucleotide polymorphisms (SNPs) or insertions/deletions (INDELs) with an allele frequency ≥ 5% having a total depth of coverage >100x were manually reviewed and interpreted.

### Statistical analysis

Continuous variables were reported as mean and standard deviation or median and interquartile range and were compared by using the Student *t* test or Mann Whitney U test. Categorical variables were reported as frequency (%) and were compared by either a 2-tailed Fisher exact test or Pearson χ^2^ test, where appropriate. Multivariable modeling was not performed due to the small number of events and small numbers of patients with identified genetic variants. Progression free survival (PFS) was defined as the time from FNA to any objective evidence of disease progression or death, whichever occurred firstly. PFS and overall survival (OS) curves were estimated with the Kaplan Meier method and were compared with the log rank test. All tests were 2-sided, with *P* ≤ .05 as the criterion standard for determining significance. The statistical software package JMP Version 11 (SAS Institute, Cary, NC) and MedCalc version 12 (MedCalc Software, Mariakerke, Belgium) were used for statistical analysis.

## SUPPLEMENTARY MATERIALS FIGURE AND TABLES



## Abbreviations

ATRX: Alpha Thalassemia/Mental Retardation Syndrome X-Linked
DAXX: Death-Domain Associated Protein
EUS FNA: Endoscopic Ultrasound Fine Needle Aspiration
pNET: Pancreas Neuroendocrine Tumor
MEN1: Menin 1 gene
mTOR: Mammalian target of rapamycin
NGS: Next generation sequencing
ROSE: Rapid on site evaluation
TSC2: Tuberous sclerosis 2 gene
VUS: Variant of unknown significance
WHO: World Health Organization
WT: Wild type (no somatic mutation detected)
