# Supplementary material for: Assessment of pancreatic neuroendocrine tumor cytologic genotype diversity to guide personalized medicine using a custom gastroenteropancreatic next-generation sequencing panel
Source: Oncotarget. 2017 Jun 28;8(55):93464–75. doi: 10.18632/oncotarget.18750 (PMC5706811; doi:10.18632/oncotarget.18750)
Supplement: Supplementary file 1 [file oncotarget-08-93464-s001.pdf]

Assessment of pancreatic neuroendocrine tumor cytologic genotype diversity to guide personalized medicine using a custom gastroenteropancreatic next-generation sequencing panel

SUPPLEMENTARY MATERIALS

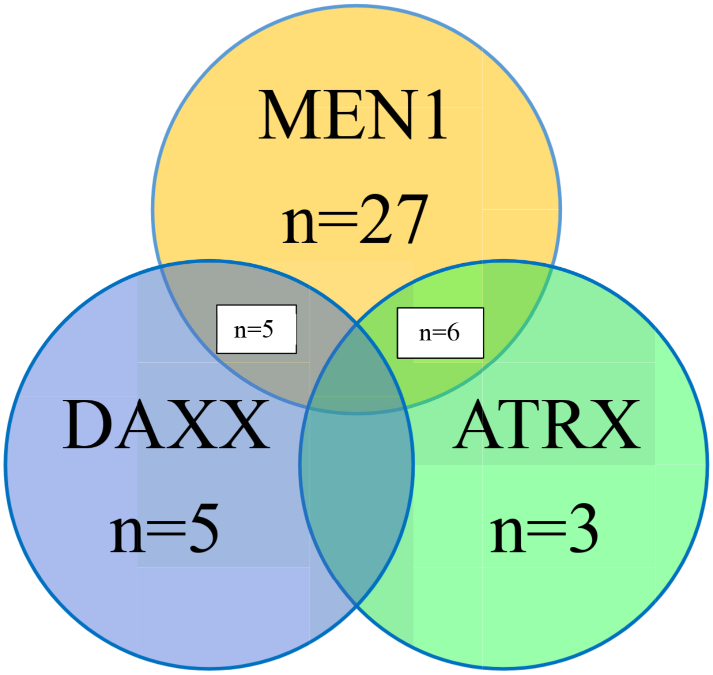

Supplementary Figure 1: Distribution of MEN1, DAXX and ATRX primary tumor variants.

Supplementary Table 1: Custom 15 gene gastroenteropancreatic panel specifics

locus	**chrom**	**target regions**	**target bases**	**bases covered**	**% bases covered**	**amplicons**	**size min**	**size avg**	**size max**
NRAS	chr1	3	570	570	100	8	130	145	150
RET	chr10	12	2,363	2,124	89.9	30	124	157	200
PTEN	chr10	9	1572	1520	196.4	26	131	147	200
HRAS	chr11	3	570	306	53.7	6	123	156	199
MEN1	chr11	9	2,208	1,995	90.4	29	125	153	195
KRAS	chr12	3	570	570	100	9	130	142	150
RB1	chr13	27	3,867	3,690	95.4	64	130	146	191
TSC2	chr16	42	7377	6584	92.8	103	120	156	200
TP53	chr17	11	1,743	1,742	99.9	30	120	146	178
VHL	chr3	3	762	700	91.9	9	133	147	186
CTNNB1	chr3	1	268	268	100	4	133	142	150
PIK3CA	chr3	8	1,863	1,837	98.6	29	130	146	150
DAXX	chr6	8	2,596	2,596	100	40	120	145	177
BRAF	chr7	3	495	416	84	5	130	156	200
ATRX	chrX	36	8,969	8,969	100	141	130	145	161
Totals		178	35,793	33,887	94.7	533	120	149	200

Supplementary Table 2: Primary pancreas spectrum vs. pancreas liver met genetic variant spectrum

	Gene	Primary pNET n=90 patients	pNET liver n=32 patients	P value
1.	MEN1	38 (42.2 %)	11 (34.4%)	0.5305
2.	DAXX	10 (11.1 %)	7 (21.9%)	0.1450
3.	ATRX	9 (10.0 %)	1 (3.1%)	0.4521
4.	TSC2	7 (7.8 %)	2 (6.3%)	0.9
5.	KRAS	3 (3.3 %)	2 (6.3%)	0.6055
6.	TP53	3 (3.3 %)	5 (15.6%)	**0.0287**
7.	RB1	2 (2.2 %)	0 (0%)	0.9
8.	CTNNB1	2 (2.2 %)	0 (0%)	0.9
9.	RET	1 (1.1 %)	0 (0%)	0.9
10.	PTEN	1 (1.1 %)	2 (6.3%)	0.1680
11.	HRAS	1 (1.1 %)	0 (0%)	0.9
12.	PIK3CA	1 (1.1 %)	0 (0%)	0.9
13.	VHL	0 (0%)	1 (3.1%)	0.2623

°NRAS and BRAF not include as no identified variants.

Supplementary Table 3: Pathogenic alterations and Variants of Unknown Significance (VUS) breakdown

	Gene	No. of variants identified	No. of pathogenic alterations	No. of VUS
1.	MEN1	43	43	0
2.	DAXX	10	8	2
3.	ATRX	12	6	6
4.	TSC2	7	5	2
5.	KRAS	3	3	0
6.	TP53	3	3	0
7.	RB1	2	2	0
8.	CTNNB1	2	2	0
9.	RET	1	0	1
10.	PTEN	1	1	0
11.	HRAS	1	0	1
12.	PIK3CA	1	0	1
13.	NRAS	0	0	0
14.	VHL	0	0	0
15.	BRAF	0	0	0

Supplementary Table 4: MEN1mutation nomenclature and consequence

	Number of Variants	Current Mutation Nomenclature	Consequence
1.	1	MEN1 c.978delC, p.Tyr326*	frameshift variant
2.	1	MEN1 c.928G>C, p.Gly310Arg	missense variant
3.	1	MEN1 c.872T>C, p.Leu291Pro	missense variant
4.	1	MEN1 c.798+1G>A	splice site variant
5.	1	MEN1 c.76G>T, p.Glu26*	nonsense
6.	1	MEN1 c.740C>T, p.Ala247Val	missense variant
7.	1	MEN1 c.729delC, p.Phe243Leufs*43	frameshift variant
8.	2	MEN1 c.669+3A>G	splice site variant
9.	1	MEN1 c.628delG, p.Asp210Thrfs*19	frameshift variant
10.	1	MEN1 c.608G>A, p.Trp203*	nonsense
11.	1	MEN1 c.56_68delinsGG, p.Val19Glyfs*94	frameshift variant
12.	1	MEN1 c.551A>T, p.Glu184Val	missense variant
13.	1	MEN1 c.531delT, p.Asp177Glufs*13	frameshift variant
14.	1	MEN1 c.518T>C, p.Leu173Pro	missense variant
15.	1	MEN1 c.511C>T, p.Gln171*	nonsense
16.	1	MEN1 c.509G>A, p.Cys170Tyr	missense variant
17.	1	MEN1 c.493_505delGCTGTGGTTGGGG, p.Ala165Profs*21	frameshift variant
18.	1	MEN1 c.493_505del, p.Ala165Profs*21	frameshift variant
19.	1	MEN1 c.488C>A, p.Ala163Asp	missense variant
20.	1	MEN1 c.402delC, p.Phe134Leufs*20	frameshift variant
21.	1	MEN1 c.381_382delCA, p.Asn127Lysfs*57	frameshift variant
22.	2	MEN1 c.360_373delGGTCTCCGATGTCA, p.Lys120Asnfs*60	frameshift variant
23.	1	MEN1 c.322C>T, p.Arg108*	nonsense
24.	1	MEN1 c.2T>G	start loss
25.	1	MEN1 c.248delT, p.Leu83Argfs*36	frameshift variant
26.	1	MEN1 c.207delC, p.Asp70Thrfs*49	frameshift variant
27.	1	MEN1 c.1675C>T, p.Gln559*	nonsense
28.	1	MEN1 c.1372delinsGTCGGG, p.Gln458ValfsX7	Frameshift variant
29.	1	MEN1 c.1372C>T, p.Gln458*	nonsense
30.	1	MEN1 c.133G>T, p.Glu45*	nonsense
31.	1	MEN1 c.1282_1297del, p.Trp428Profs*17	frameshift variant
32.	1	MEN1 c.1277_1278delGCinsAA, p.Cys426*	nonsense
33.	1	MEN1 c.1267G>T, p.Asp423Tyr	missense variant
34.	3	MEN1 c.1258C>T, p.Arg420*	nonsense
35.	1	MEN1 c.1192C>T, p.Gln398*	nonsense
36.	2	MEN1 c.117_128delGGTGCTGGGCTT, p.Leu39_Gly42del	In frame deletion
37.	1	MEN1 c.1109_1110delTT, p.Phe370*	frameshift variant
38.	1	MEN1 c.1046C>G, p.Thr349Arg	missense variant

Supplementary Table 5: DAXX mutation nomenclature and consequence

	Number of variants	Current Mutation Nomenclature	Consequence
1.	1	DAXX c.344G>A, p.Arg115Gln	missense variant
2.	1	DAXX c.1898G>A, p.Arg633Gln	missense variant
3.	1	DAXX c.905_906del, p.Leu302Argfs*41	frameshift variant
4.	1	DAXX c.825del, p.Asn276Thrfs*18	frameshift variant
5.	1	DAXX c.660_664del, p.Ala221Profs*9	frameshift variant
6.	1	DAXX c.61C>T, p.Gln21*	nonsense
7.	1	DAXX c.2218del, p.Asp740Ilefs*26	frameshift variant
8.	1	DAXX c.1884dup, p.Cys629Leufs*29	frameshift variant
9.	2	DAXX c.1884del, p.Cys629Alafs*16	frameshift variant

*n=7 (70%) frameshift variant; n=2 (20%) missense variant; n=1 (10%) stop gained.

Supplementary Table 6: ATRX mutation nomenclature and consequence

	Number of variants	Current Mutation Nomenclature	Consequence
1.	2	ATRX c.6835G>T, p.Glu2279*	nonsense
2.	2	ATRX c.6825G>A, p.Trp2275*	nonsense
3.	1	ATRX c.6577C>G, p.Gln2193Glu	missense variant
4.	1	ATRX c.5186C>A, p.Ser1729Tyr	missense variant
5.	1	ATRX c.3943+1_3943+3delGTG	splice site variant
6.	1	ATRX c.3814G>C, p.Ala1272Pro	missense variant
7.	1	ATRX c.3700G>C, p.Val1234Leu	missense variant
8.	1	ATRX c.2919dupG, p.Ser974Glufs*3	frameshift variant
9.	1	ATRX c.2893G>A, p.Ala965Thr	missense variant
10.	1	ATRX c.2484G>C, p.Met828Ile	missense variant

[*]n=6 (50%) missense variant; n=4 (33.3%) stop gained; n=1 (8.3%) frameshift variant; n=1 (8.3%) splice site variant.

Supplementary Table 7: TSC2 mutation nomenclature and consequence

	Number of variants	Current Mutation Nomenclature	Consequence
1.	1	TSC2 c.849_850del, p.Arg283Serfs*54	frameshift variant
2.	1	TSC2 c.5260-1G>T	splice site variant
3.	1	TSC2 c.45dup, p.Lys16*	frameshift variant
4.	1	TSC2 c.436C>G, p.Leu146Val	missense variant
5.	1	TSC2 c.400G>A, p.Glu134Lys	missense variant
6.	1	TSC2 c.3298G>A, p.Val1100Met	missense variant
7.	1	TSC2 c.1080del, p.Leu361Cysfs*2	frameshift variant

* n=3 (42.9%) missense variant; n=3 (42.9%) frameshift variant; n=1 (14.3%) splice site variant.

Supplementary Table 8: Patients with stage T1N0M0 pNETs harboring adverse prognostic biomarkers with outcome

	Age (years)	Lesion size/ location	Genetic Variant	Surgery	Disease progression	Time of Disease Progression	Mortality
1.	76	12mm neck	KRAS, RB1, TP53	Whipple Procedure	Liver metastasis	0.5 years	0.97 years
2.	39	17mm neck	TSC2	Extended distal pancreatectomy	Liver metastasis	10.9 years	No
3.	68	10mm body	TSC2	No surgery	No progression	No progression	2.4 years unrelated to pNET
